# *In Vivo* Study of the Sorbicillinoid Gene Cluster in *Trichoderma reesei*

**DOI:** 10.3389/fmicb.2017.02037

**Published:** 2017-10-20

**Authors:** Christian Derntl, Fernando Guzmán-Chávez, Thiago M. Mello-de-Sousa, Hans-Jürgen Busse, Arnold J. M. Driessen, Robert L. Mach, Astrid R. Mach-Aigner

**Affiliations:** ^1^Research Area Biochemical Technology, Institute of Chemical, Environmental & Biological Engineering, Vienna, Austria; ^2^Molecular Microbiology, Groningen Biomolecular Sciences and Biotechnology Institute, University of Groningen, Groningen, Netherlands; ^3^Institute of Microbiology, University of Veterinary Medicine, Vienna, Austria

**Keywords:** sorbicillinoids, sorbicillinol, 5-hydroxyvertinolide, *Trichoderma reesei*, *Acremonium chrysogenum*, *Penicillium chrysogenum*

## Abstract

Sorbicillinoids are a diverse group of yellow secondary metabolites that are produced by a range of not closely related ascomycetes, including *Penicillium chrysogenum*, *Acremonium chrysogenum*, and *Trichoderma reesei*. They share a similarity to the name-giving compound sorbicillin, a hexaketide. Previously, a conserved gene cluster containing two polyketide synthases has been identified as the source of sorbicillin, and a model for the biosynthesis of sorbicillin in *P. chrysogenum* has been proposed. In this study, we deleted the major genes of interest of the cluster in *T. reesei*, namely *sor1*, *sor3*, and *sor4*. Sor1 is the homolog of *P. chrysogenum* SorA, which is the first polyketide synthase of the proposed biosynthesis pathway. Sor3 is a flavin adenine dinucleotide (FAD)-dependent monooxygenase, and its homolog in *P. chrysogenum*, SorC, was shown to oxidize sorbicillin and 2′,3′-dihydrosorbicillin to sorbicillinol and 2′,3′-dihydrosorbicillinol, respectively, *in vitro*. Sor4 is an FAD/flavin mononucleotide-containing dehydrogenase with an unknown function. We measured the amounts of synthesized sorbicillinoids throughout growth and could verify the roles of Sor1 and Sor3 *in vivo* in *T. reesei*. In the absence of Sor4, two compounds annotated to dihydrosorbicillinol accumulate in the supernatant and only small amounts of sorbicillinol are synthesized. Therefore, we suggest extending the current biosynthesis model about Sor4 reducing 2′,3′-dihydrosorbicillin and 2′,3′-dihydrosorbicillinol to sorbicillinol and sorbicillinol, respectively. Sorbicillinol turned out to be the main chemical building block for most sorbicillinoids, including oxosorbicillinol, bisorbicillinol, and bisvertinolon. Further, we detected the sorbicillinol-dependent synthesis of 5-hydroxyvertinolide at early time points, which contradicts previous models for biosynthesis of 5-hydroxyvertinolide. Finally, we investigated whether sorbicillinoids from *T. reesei* have a growth limiting effect on the fungus itself or on plant pathogenic fungi or on pathogenic bacteria.

## Introduction

**FIGURE 1:**
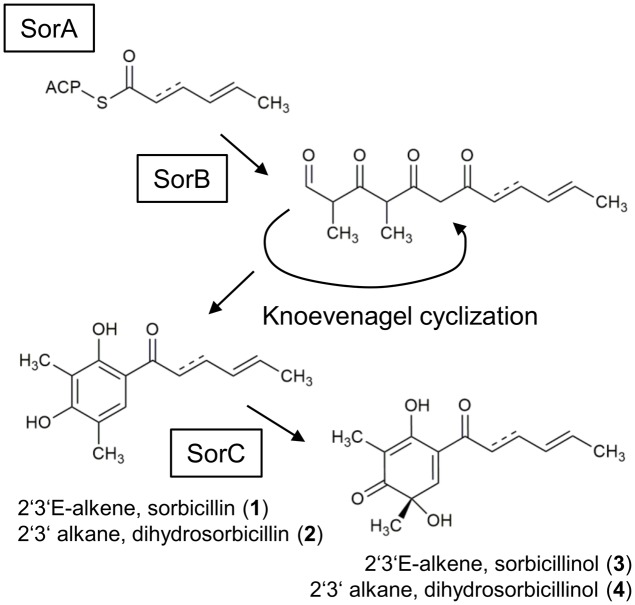
Currently suggested sorbicillinol biosynthesis pathway. The iterative PKS SorA combines three acetyl units, and the growing chain is modified by the ketoacyl reductase subunit, removing the keto-groups. The enoyl reductase subunit might be active in the second cycle, which inserts a single bond. The polyketide is handed over to the PKS SorB, which adds three more acetyl units and two methyl groups. SorB releases an aldehyde, which undergoes spontaneous cyclization, resulting in the formation of sorbicillin (**1**) or 2′,3′-dihydrosorbicillin (**2**), depending on whether the enoyl reductase subunit had been active in SorA or not. Finally, the FAD-dependent monooxygenase SorC transforms sorbicillin or 2′,3′-dihydrosorbicillin to sorbicillinol (**3**) or 2′,3′-dihydrosorbicillinol (**4**).

Sorbicillinoids are a group of yellow secondary metabolites that are produced by a range of ascomycetes, including *Penicillium* (Cram, 1948) and *Trichoderma* (Abe et al., 2000). Many of these hexaketide metabolites are highly oxygenated and have complex bicyclic and tricyclic frameworks (Harned and Volp, 2011). However, their name-giving, common feature is an apparent similarity of their core structures to sorbicillin (**Figure 1**, compound **1**). Many sorbicillinoids possess interesting bioactive properties. For instance, bisorbicillinoids have been demonstrated to have outstanding radical scavenging properties (Abe and Hirota, 2002). Additionally, trichodimerol was shown to inhibit the tumor necrosis factor-a (TNF-a) via targeting the prostaglandin H synthase-2, and thus to act anti-inflammatorily (Mazzucco and Warr, 1996; Warr et al., 1996). Further, some sorbicillinoids have been demonstrated to show antimicrobial activity (Maskey et al., 2005; Reategui et al., 2006). Zhao et al. (2017) observed anti-HIV and anti-inflammatory activities of sorbicillinoids. For detailed reviews about sorbicillinoids and their bioactive properties refer to (Meng et al., 2016) and (Harned and Volp, 2011).

The proposed biosynthesis pathway of sorbicillin in *P. chrysogenum* includes the consecutive action of the two polyketide synthases (PKS), SorA and SorB, resulting in the release of an aldehyde that undergoes spontaneous cyclization, yielding sorbicillin or 2′,3′-dihydrosorbicillin (**Figure 1**; Fahad et al., 2014). Notably, sorbicillin and 2′,3′-dihydrosorbicillin were isolated and identified from *P. chrysogenum* cultures previously (Trifonov et al., 1983). Fahad et al. (2014) demonstrated that an oxidative dearomatization of sorbicillin and 2′,3′-dihydrosorbicillin (**2**) by the flavin adenine dinucleotide (FAD)-dependent monooxygenase SorC leads to the formation of sorbicillinol (**3**), and 2′,3′-dihydrosorbicillinol (**4**) *in vitro*, respectively (**Figure 1**). Sorbicillinol is highly reactive and therefore considered to be the main building block for the formation of bicyclic sorbicillinoids such as bisorbicillinol and trichodimerol (Harned and Volp, 2011).

**FIGURE 2:**
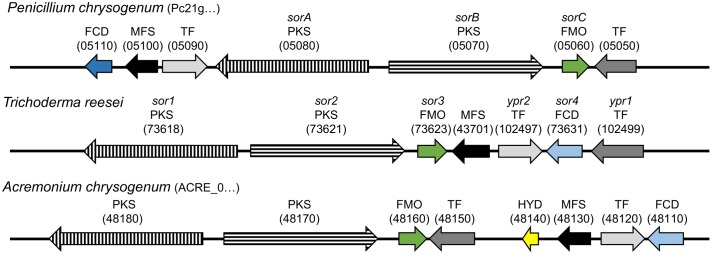
Genomic organization of the sorbicillin cluster in *P. chrysogenum*, *T. reesei*, and *A. chrysogenum.* The arrows indicate the genes belonging to the sorbicillin cluster in the depicted fungi. Arrows with the same filling (color/pattern) represent homologs. The FCD Pc21g05110 shares only the same domain architecture with homologs FCDs *sor4* and ACRE_048110, which is indicated by the distinction between dark and light blue. Orientation, distance, and size are to scale and comparable. The beginning of a protein IDs is given in brackets after the species name and the variable part of the ID in brackets above the respective arrow. FCD, FAD/FMN-containing dehydrogenase; MFS, transporter of the multifacilitator superfamily; TF, transcription factor; PKS, polyketide synthase; FMO, FAD-dependent monooxygenase; HYD, hydrolase.

In *P. chrysogenum*, the genes encoding for the two PKS, SorA and SorB, and the FAD-dependent monooxygenase SorC are part of a gene cluster (Salo et al., 2016), which is also present in a range of not closely related ascomycetes, including *T. reesei* and *Acremonium chrysogenum* (Martinez et al., 2008; Terfehr et al., 2014; Derntl et al., 2016; **Figure 2**). The core set of the gene cluster consists of genes encoding for two PKS, an FAD-dependent monooxygenase, a transporter, and two Gal4-like transcription factors. Additionally, some auxiliary genes can be present (**Figure 2**). *P. chrysogenum* Pcg21g05110, *T. reesei* protein ID 73631 (*sor4*), and *A. chrysogenum* ACRE_048110 are FAD/flavin mononucleotide (FMN)-containing dehydrogenases containing a “berberine and berberine like” domain according to a NCBI conserved domain search (Marchler-Bauer et al., 2015). Despite the similar domain architecture, only *T. reesei sor4* and *A. chrysogenum* ACRE_048110 appear to be orthologous according to a DELTA-BLAST analysis (Boratyn et al., 2012). Further, the hydrolase ACRE_048140 is only present in *A. chrysogenum*; ACRE_048140 is related to *Monascus ruber ctnB*/*citA* which is considered to support the respective PKS in the citrinin biosynthesis pathway (He and Cox, 2016).

Recently, we verified that the PKS SorA is essential for the sorbicillinoid biosynthesis in *P. chrysogenum* (Salo et al., 2016). In *T. reesei*, which is studied and industrially applied for its outstanding protein secretion capabilities of plant cell wall-degrading enzymes (Peterson and Nevalainen, 2012), we identified the main regulator of the sorbicillinoid gene cluster (Derntl et al., 2016). The deletion of this transcription factor, Yellow pigment regulator 1 (Ypr1, **Figure 2**), abolishes the synthesis of the yellow sorbicillinoids and the expression of all genes of cluster, except of *sor4*.

In this study, we deleted the cluster genes encoding for the first PKS, the FAD-dependent monooxygenase, and the FAD/FMN-containing dehydrogenase in *T. reesei*. Further, we expressed the *citA*/*ctnB*-similar hydrolase from *A. chrysogenum* in *T. reesei*. The strains were investigated together with a *ypr1* deletion strain and a *ypr1* overexpression strain regarding their growth and their yellow pigment synthesis behavior. Further, the presence and the abundance of sorbicillinoids in their supernatants were measured, allowing us to extend the existing model for the biosynthesis pathway of sorbicillinoids. Additionally, we assayed the influence of the sorbicillinoids on the growth of other fungi and bacteria and on the confrontation behavior of *T. reesei* in presence of plant pathogenic fungi.

## Materials and Methods

### Fungal Strains and Cultivation Conditions

All *T. reesei* strains (**Table 1**) were maintained on malt extract agar at 30°C. Uridine was added to a final concentration of 5 mM if applicable. Mandels–Andreotti (MA) medium (Mandels, 1985) without peptone containing 1% (w/v) D-glucose was used as minimal medium for selections after fungal transformations. For cultivation on D-glucose, *T. reesei* was grown in 300 ml MA medium containing 1% (w/v) D-glucose 30°C on a rotary shaker at 180 rpm. Mycelia and supernatants were separated by filtration through Miracloth (EMD Millipore, part of Merck KGaA, Darmstadt, Germany). Mycelia were dried at 80°C overnight for biomass determination and supernatants were stored at -20°C. Mycelia for RNA isolation and transcript analysis were harvested after 36 h cultivation and stored over liquid nitrogen. *Fusarium oxysporum* Foc4 (TUCIM 4812), *Alternaria alternata* TUCIM 3737, *Rhizoctonia solani* TUCIM 3753, and *Thanatephorus cucumeris* (*Botrytis cinerea*) TUCIM 4679 were maintained on potato dextrose agar at room temperature (approximately 22°C).

**Table 1:** *Trichoderma reesei* strains used throughout this study.

Strain	Abbreviation	Source
QM6aΔ*tmus53*	QM6a	Steiger et al., 2011
QM6aΔ*tmus53*Δ*pyr4*	Δ*pyr4*	Derntl et al., 2015
QM6aΔ*tmus53*Δ*ypr1*	Δ*ypr1*	Derntl et al., 2016
QM6aΔ*tmus53*Re*ypr1*	Re*ypr1*	Derntl et al., 2016
QM6aΔ*tmus53*Δ*sor1*	Δ*sor1*	This study
QM6aΔ*tmus53*Δ*sor3*	Δ*sor3*	This study
QM6aΔ*tmus53*Δ*sor4*	Δ*sor4*	This study
QM6aΔ*tmus53*p*sor3*::ACRE_048140 (*pyr4*)	A4814	This study

### Bacterial Strains and Cultivation Conditions

*Escherichia coli* Top10 (Invitrogen, part of Thermo Fisher Scientific Inc., Waltham, MA, United States) was used for all cloning purposes throughout this study and maintained on LB at 37°C. If applicable, ampicillin was added to a final concentration of 100 μg/ml. *Staphylococcus aureus* DSM 20231^T^, a methicillin-resistant *S. aureus* (MRSA) strain, an *E. coli* strain, and a multi-resistant *Acinetobacter baumannii* strain (the latter three are isolates from the clinical diagnostics unit at the Institute of Microbiology, Veterinary University Vienna) were grown on Peptone Yeast Extract (PYE) agar [0.3% (w/v) peptone from casein, 0.3% (w/v) yeast extract, 1.5% (w/v) agar–agar, pH 7.2] and used to test sensibility for sorbicillinoids.

### Plasmid Constructions

Polymerase chain reactions (PCRs) for cloning purposes were performed with Q5 High-Fidelity DNA Polymerase (New England Biolabs, Ipswich, MA, United States) according to the manufacturer’s instructions. All used primers are listed in Supplementary Table S1. PCR products were cloned into *Eco*RV-digested pJET1.2 (Thermo Scientific, part of Thermo Fisher Scientific Inc., United States), and after verification of the PCR products by sequencing (Microsynth, Balgach, Switzerland), they were released for subsequent cloning purposes by digestion with suitable restriction endonucleases.

For the construction of the plasmids pCDΔsor1, pCDΔsor3, and pCDΔsor4 the 5′- and 3′-flanking regions of the respective genes were amplified by PCR using chromosomal DNA of *T. reesei* QM6a as template and corresponding primers given in Supplementary Table S1. Consecutively, corresponding flanking regions were inserted into pJET-pyr4 (Derntl et al., 2016) using the restriction enzymes indicated in the primer names.

For the construction of pRP4-ACRE_048140_ex_ the promoter of *sor3* was amplified by PCR using chromosomal DNA of *T. reesei* QM6a as template and the primers P*_sor3_*_fwd-BspEI and P*_sor3_*_rev-SpeI and inserted into pCD-RPyr4T (Derntl et al., 2015) using *Bsp*EI and *Spe*I. The coding sequence of ACRE_048140 was amplified by PCR using chromosomal DNA of *A. chrysogenum* ATCC 11550 as template and the primers ACRE_048140_fwd-SpeI and ACRE-048140_rev-BamHI and subsequently inserted into the latter plasmid using *Spe*I and *Bam*HI.

### Fungal Transformation

The protoplast transformation of *T. reesei* was performed as described earlier (Gruber et al., 1990). Typically, 30 μg of linearized plasmid DNA of the plasmids pCDΔsor1, pCDΔsor3, pCDΔsor4, or pRP4-ACRE_048140_ex_ (in 15 μl sterile ddH_2_O) was used for the transformation of 10^7^ protoplasts of the strain Δ*pyr4*. For selection for prototrophy, 100 μl to 2 ml of the transformation reaction were added to 20 ml melted, 50°C warm minimal medium agar containing 1.2 M sorbitol. This mixture was poured into sterile petri dishes. The plates were incubated at 30°C for 3–5 days until colonies were visible. Candidates were subjected to homokaryon selection by spore streak-outs on selection medium plates to obtain stable, homokaryotic strains.

### Genotype Testing

Chromosomal DNA was isolated from mycelium by grinding in liquid nitrogen followed by a phenol/chloroform extraction (Gruber et al., 1990). RNA was degraded using RNaseA (Thermo Scientific). DNA was precipitated with isopropanol, washed with 70% ethanol, and dissolved in ddH_2_O. For testing the genotype, 10 ng of chromosomal DNA were used as template in a 25-μl PCR using GoTaq^®^ G2 polymerase (Promega, Madison, WI, United States) according to the manufacturer’s instructions. All used primers are listed in Supplementary Table S1. For subsequent agarose gel electrophoresis of DNA fragments a GeneRuler 1 kb DNA Ladder (Thermo Scientific, United States) was applied for estimation of the fragment size.

### RNA Isolation and RT-PCR

Approximately 20 mg of harvested mycelia were homogenized in 1 ml of peqGOLD TriFast DNA/RNA/protein purification system reagent (PEQLAB Biotechnologie, Erlangen, Germany) using a FastPrep FP120 BIO101 ThermoSavant cell disrupter (Qbiogene, Carlsbad, CA, United States). RNA was isolated according to the manufacturer’s instructions, and the concentration was measured using the NanoDrop 1000 (Thermo Scientific). Synthesis of cDNA from mRNA was carried out using the RevertAid^TM^ H Minus First Strand cDNA Synthesis Kit (Thermo Scientific) according to the manufacturer’s instructions.

Reverse transcription polymerase chain reactions (RT-PCRs) were performed in a Mastercycler^®^ ep realplex 2.2 system (Eppendorf, Hamburg, Germany). All reactions were performed in triplicates. The amplification mixture (final volume 25 μl) contained 12.5 μl 2 × iQ SYBR Green Mix (Bio-Rad), 100 nM forward and reverse primer, and 2.5 μl cDNA (diluted 1:100) as template. All used primers are listed in Supplementary Table S1. Cycling conditions and control reactions were performed as described earlier (Steiger et al., 2010).

### Metabolite Analysis

Culture supernatants were filtered using a 2 μm-pore polytetrafluoroethylene (PTFE) syringe filter. Liquid chromatography–mass spectrometry (LC-MS) analysis was performed in an Accella 1250 LC system coupled with the benchtop ES-MS Orbitrap Exactive (Thermo Fisher Scientific, United States) as described earlier (Salo et al., 2016). Reserpine is used as an internal standard. The differential analysis was done using the Thermo Scientific 205 SIEVE software. The response ratio indicates the fold-change of the compound detected in relation to reserpine. The metabolite analysis was performed from single representative biological samples in technical duplicates. Reserpine (Sigma–Aldrich, United States) was used as internal standard.

### Fungal Plate Confrontation Assays

*Trichoderma reesei* strains were cultivated in MA medium containing 1% (w/v) D-glucose at 30°C at 180 rpm for 48 h. The cultivation supernatant was filtered using a 2-μm-pore syringe filter, mixed 1:1 (v/v) with melted potato dextrose agar that contained additional agar–agar, and poured into sterile petri dishes. The resulting plates were inoculated with agar pieces overgrown with plant pathogenic fungi, which were pre-grown on potato dextrose agar, and were incubated at room temperature. For fungal confrontation assays, all strains were pre-grown on potato dextrose agar at room temperature. Overgrown agar pieces of a *T. reesei* strain and a plant pathogenic strain, respectively, were transferred to opposite sides of a single potato dextrose agar plate and the plates were incubated at room temperature.

### Agar Diffusion Assay

Suspensions of the pathogenic bacterial strains were spread on PYE agar plates. Different filter disks each soaked with 30 μl of each supernatant obtained from 48 h of cultivation of *T. reesei* strains and, as control, unconditioned MA medium were placed circularly on each agar plate and incubated at 37°C. The results were evaluated after 12, 36, and 50 h.

## Results

### Construction of Recombinant *T. reesei* Strains to Characterize the Sorbicillin Cluster

In order to get insights into the biosynthesis of sorbicillinoids in *T. reesei*, we constructed a set of corresponding strains. We deleted the homolog of *sorA*, *sor1* (**Figure 2**), which we expected to result in a complete abolishment of the biosynthesis pathway (**Figure 1**). We also deleted the homolog of *sorC*, *sor3* (**Figure 2**), which we expected to interrupt the pathway prior to the oxidative dearomination of sorbicillin and 2′,3′-dihydrosorbicillin (**Figure 1**). Further, we deleted *sor4*, and inserted ACRE_048140 (**Figure 2**); both genes were not characterized yet. All gene deletions were performed via homologous recombination using reestablishment of *pyr4* as marker (Supplementary Figure S1A). The expression cassette for ACRE_048140 was inserted into the *pyr4* locus as described earlier (Derntl et al., 2015; Supplementary Figure S2A). The genomic manipulations were confirmed by PCR analyses (Supplementary Figures S1B, S2B). Further, the absence of *sor1*, *sor3*, and *sor4* transcripts in the corresponding deletions strains and the presence of ACRE_048140 transcripts in the strain A4814 were confirmed by RT-PCR. The *ypr1* transcript was used as positive control for expression of the whole cluster (Supplementary Figures S1C, S2C).

**FIGURE 3:**
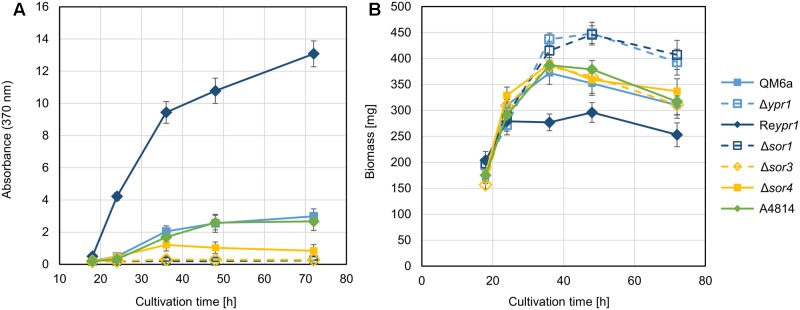
Phenotypic comparison of the recombinant *T. reesei* strains. The indicated *T. reesei* strains were cultivated on glucose for the indicated periods, and absorbance at 370 nm of the supernatants **(A)** and biomass accumulation (dry weight) **(B)** were determined. The gives values are the mean of three replicates. The error bars represent the standard deviations.

Next, we cultivated the obtained strains Δ*sor1*, Δ*sor3*, Δ*sor4*, and A4814 together with the strain QM6a, which contains the wild-type version of the gene cluster, the *ypr1* deletion strain Δ*ypr1*, and the *ypr1* overexpression strain Re*ypr1* on glucose. As Ypr1 is the main regulator of the sorbicillin cluster, Δ*ypr1* is deficient in synthesis of sorbicillinoids, and Re*ypr1* produces high amounts of sorbicillinoids (Derntl et al., 2016). We monitored growth and biosynthesis of the yellow sorbicillinoids by measuring biomass accumulation and absorbance at 370 nm, respectively. As expected, the supernatants of Δ*ypr1*, Δ*sor1*, and Δ*sor3* did not turn yellow (**Figure 3A**), but we could measure high absorbance for Re*ypr1* (**Figure 3A**). A4814 had the same phenotype as QM6a, whereas Δ*sor4* secreted less yellow pigments than QM6a at time points after 36 h (**Figure 3A**). Further, we observed that the strains Δ*ypr1* and Δ*sor1* produced more biomass than all the other strains (**Figure 3B**). *Vice versa*, Re*ypr1* produced the lowest amount of biomass. The remaining strains grew equally well (**Figure 3B**). On a first glance, it appears as if the synthesis of the sorbicillinoids impairs growth of *T. reesei*.

### Sorbicillinol Is the Main Product of the Biosynthesis Pathway

We were interested in what kind of sorbicillinoids were produced and accumulated in the recombinant strains. Consequently, we performed an LC-MS analysis of the culture supernatants from the above-described growth experiment on glucose (**Figure 3**). We used samples from early, middle, and late time points (24, 36, and 72 h). All compounds that were detected in the supernatant of any strain that produces sorbicillinoids (QM6a, Re*ypr1*, Δ*sor4*, or A4814), but not in strains that are deficient in sorbicillinoid synthesis (Δ*ypr1* and Δ*sor1*) are listed in **Table 2**. In the supernatant of strain Δ*sor3*, in which biosynthesis is assumed to be interrupted after sorbicillin generation (**Figure 1**), only traces of sorbicillinol were detected (**Table 3**). We consider them to be the result of chemical conversions and not of enzymatic activity. Further, the two compounds J_207 and F193 were also present in Δ*sor3* (**Table 3**). We suggest that J_207 and F193 might be degradation products of sorbicillin or precursors that occur during sorbicillin biosynthesis, because they have lower masses than sorbicillin (**Table 2**). Therefore, we did not include them in further data interpretations. However, we also detected a series of compounds containing up to nine nitrogen atoms in the sorbicillinoid producing strains (**Table 2**). We consider these compounds (D_293, C_309, E_333, M_479, A_556, and S_657) to be the result of a sponanteous chemical reaction of sorbicillinoids with urea and/or ammonium ions in the medium. Therefore, we decided to omit also these compounds in further data interpretations.

**Table 2:** Compounds detected in the supernatants of the strains *T. reesei* QM6a, Re*ypr1*, A4814, or Δ*sor4*.

Compound ID	Acquired [M+H]^+^	RT (min)	Formular	Annotated metabolite(s)
F_193	193.0862	20.41	C11H12O3	Unknown
J_207	207.1017	24.02	C12H14O3	Unknown
**N_233^1^**	**233.1174**	**32.09**	**C14H16O3**	**Sorbicillin**
**G_249**	**249.1123**	**20.89**	**C14H16O4**	**Sorbicillinol^2^**
**H_249**	**249.1123**	**22.19**	**C14H16O4**	**Sorbicillinol^2^**
**F_251**	**251.1279**	**19.74**	**C14H18O4**	**Dihydrosorbicillinol**
**J_251**	**251.1279**	**24.07**	**C14H18O4**	**Dihydrosorbicillinol**
**F_265**	**265.1071**	**19.91**	**C14H16O5**	**Oxosorbicillinol/Epoxysorbicillinol**
**L_265**	**265.1071**	**26.65**	**C14H16O5**	**Oxosorbicillinol/Epoxysorbicillinol**
**B_267**	**267.1228**	**15.37**	**C14H18O5**	**5-hydroxyvertinolide**
D_293	293.1495	16.73	C15H20O4N2	Unknown
**K_307**	**307.1543**	**25.91**	**C17H22O5**	Unknown
C_309	309.1441	15.61	C15H20O5N2	Unknown
E_333	333.1444	18.93	C16H14N9	Unknown
M_479	479.2064	29.54	C27H24O2N7	Unknown
**M_497**	**497.2166**	**29.63**	**C28H32O8**	**Bisorbicillinol**
**R_513**	**513.2117**	**34.15**	**C28H32O9**	**Bisvertinolon**
**P_515**	**515.2274**	**33.16**	**C28H34O9**	**Dihydrobisvertinolone**
**B_517**	**517.243**	**15.37**	**C28H36O9**	**Tetrahydrobisvertinolone**
A_556	556.1957	14.39	C16H29O13N9	Unknown
S_657	657.2694	36.75	C22H40O15N8	Unknown

^1^*Compounds highlighted in bold were considered for subsequent data interpretations. ^2^We consider these two compounds to be identical and refer to them as “sorbicillinols”*.

**Table 3:** Abundance of the compounds detected in the supernatant of *T. reesei* Δ*sor3*.

Compound	RR^1^ after 24 h	RR after 36 h	RR after 72 h
G_249 + H_249 (sorbicillinols)	0	0.07	0.03
J_207	0.00	0.00	0.02
F_193	0.01	0.43	0.19

^1^*Response ratio (see section “Materials and Methods”)*.

Next, we grouped the remaining metabolites (given in bold letters in **Table 2**) into three categories, i.e., (i) metabolites that occur in elevated levels at early time points of cultivation, (ii) metabolites that occur in elevated levels only in Δ*sor4*, and (iii) metabolites that occur in elevated levels at late time points of cultivation.

**FIGURE 4:**
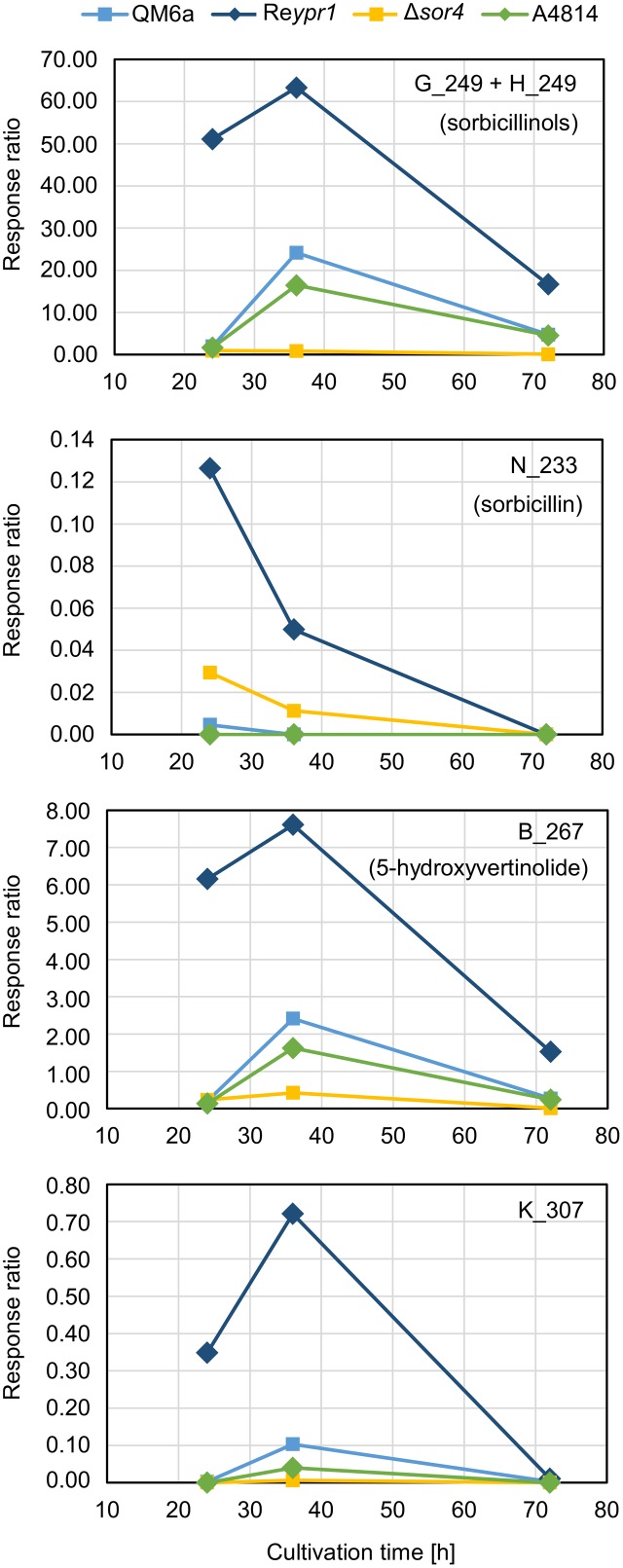
Abundance of compounds that are secreted early into the supernatant. The indicated *T. reesei* strains were cultivated on glucose in three replicates (compare **Figure 3**) for the indicated periods. A representative sample for each time point and strain was analyzed by HPLC-MS in technical duplicates. The quantities of the indicated compounds are given as response ratios (i.e., the fold-change of the compound detected in relation to the internal standard).

First, we had a detailed look on the presence and amounts of metabolites in QM6a, which is wild-type regarding the sorbicillinoids synthesis. The main early compound is sorbicillinol (**Figure 4**) or to be more precisely, sorbicillinols, because we detect two compounds with the same mass that have very similar retention times (**Table 2**). We consider them to be identical. Further, we detect small amounts of sorbicillin in the beginning (**Figure 4**). These findings are in concordance with the previously suggested biosynthesis pathway in *P. chrysogenum*, which claims that sorbicillin is oxidized to sorbicillinol (**Figure 1**). Further, we also detected 5-hydroxyvertinolide and the compound K_307 in the early stages of cultivation (**Figure 4**). The strain A4814 produced nearly the same amounts of all the early compounds as QM6a (**Figure 4**). We detected higher amounts of all early metabolites in the *ypr1* overexpression strain Re*ypr1* compared to QM6a (**Figure 4**). In Δ*sor4* we detected substantially lower amounts of sorbicillinols, 5-hydroxyvertinolide, and the compound K_307 than in QM6a, but slightly higher amounts of sorbicillin (**Figure 4**).

### Deletion of *sor4* Leads to Accumulation of Dihydrosorbicillinol

**FIGURE 5:**
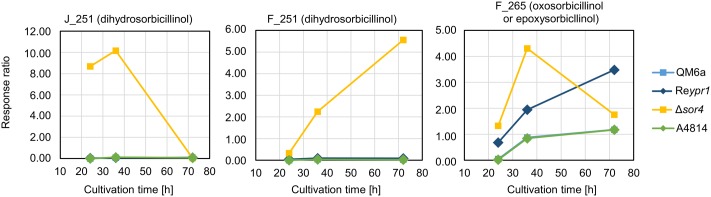
Abundance of compounds that accumulate in the supernatant over time. The indicated *T. reesei* strains were cultivated on glucose in three replicates (compare **Figure 3**) for the indicated periods. A representative sample for each time point and strain was analyzed by LC-MS in technical duplicates. The quantities of the indicated compounds are given as response ratios (i.e., the fold-change of the compound detected in relation to the internal standard).

In the Δ*sor4* strain, we observed high amounts of two compounds that were annotated to dihydrosorbicillinol (**Table 2**). The compound J_251 was predominantly detected at early time points, while F_251 accumulated throughout growth (**Figure 5**). Notably, these compounds were not detected in any other strain. We also found higher amounts of F_265, which was annotated to oxosorbicillinol or epoxysorbicillinol (**Table 2**), in strain Δ*sor4* compared to the other strains after 24 and 36 h of cultivation (**Figure 5**).

### Sorbicillinol Is the Building Block for the Other Sorbicillinoids

**FIGURE 6:**
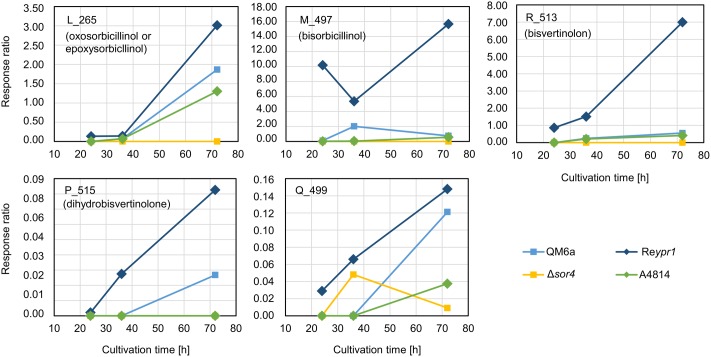
Abundance of compounds that are elevated in the supernatant of strain Δ*sor4.* The indicated *T. reesei* strains were cultivated on glucose in three replicates (compare **Figure 3**) for the indicated periods. A representative sample for each time point and strain was analyzed by LC-MS in technical duplicates. The quantities of the indicated compounds are given as response ratios (i.e., the fold-change of the compound detected in relation to the internal standard).

Next, we analyzed the abundance and occurrence of the late sorbicillinoids (**Figure 6**). We detected disorbicillinol, bisvertinolon, and two compounds that were annotated to oxosorbicillinol or epoxysorbicillinol, and traces of a dihydrobisvertinolone in QM6a (**Figure 6**). In Re*ypr1*, we measured higher levels of these late sorbicillinoids (**Figure 6**). Notably, Re*ypr1* also produces higher amounts of sorbicillinols at the early and middle time points (**Figure 4**). *Vice versa*, in A4814, lower amounts of sorbicillinols were detected than in QM6a after 36 h (**Figure 4**), and also lower amounts of the late sorbicillinoids (**Figure 6**). In Δ*sor4*, which produces very small amounts of sorbicillinols (**Figure 4**), we detected none of the late sorbicillinoids (**Figure 6**). This suggests that the late sorbicillinoids are the products of chemical and/or enzymatical conversion of sorbicillinol. Interestingly, the late metabolite Q_499 is found in small amounts in all strains (**Figure 6**). Its empirical formula suggests that is an unknown bicyclic sorbicillinoid (**Table 2**).

### The Sorbicillinoids of *T. reesei* Restrict Growth of Pathogenic Fungi But Not of Bacteria

**FIGURE 7:**
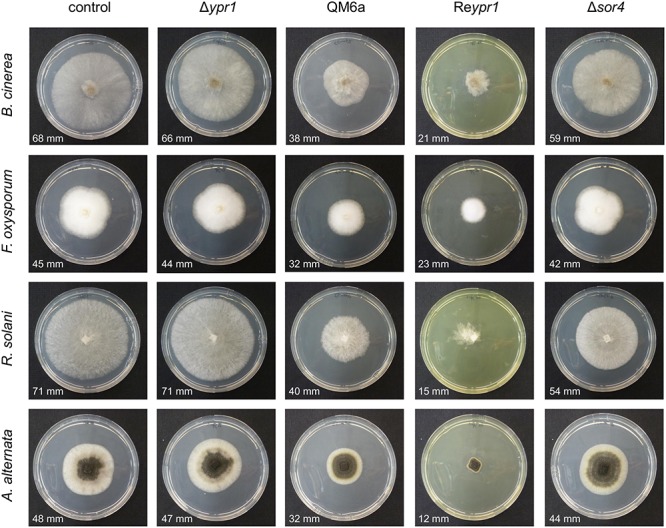
Influence of *T. reesei* sorbicillinoids on growth of plant pathogenic fungi. The *T. reesei* strains (indicated on top) were cultivated on glucose for 48 h and the resulting culture supernatants were filtered, mixed 1:1 (v/v) with potato dextrose agar, and poured into petri dishes. As a control, medium that was not inoculated with any *T. reesei* strain was used. The plant pathogenic fungi (indicated on the left) were inoculated on these plates and incubated at room temperature. Pictures were taken after 3 days in the case of *R. solani* and *B. cinerea*, and after 5 days in the case of *F. oxysporum* and *A. alternata*. The diameters of the growth halos are provided.

The cultivation experiment on glucose pointed toward a growth limiting effect of sorbicillinoids on *T. reesei*. Consequently, we were interested, whether these metabolites might also influence growth of plant pathogenic fungi. To test this, supernatants from 48 h of cultivation of the *T. reesei* strains QM6a, Δ*ypr1*, Re*ypr1*, and Δ*sor4* were filtered and added to cultivation medium in order to cast plates containing the sorbicillinoids. On these plates, the plant pathogenic fungi *A. alternata*, *B. cinerea*, *F. oxysporum*, and *R. solani* were inoculated. Here we observed a clear growth impairment of all four tested fungi caused by the secreted sorbicillinoids of *T. reesei* (**Figure 7**). On plates containing the supernatant of Δ*ypr1*, the four fungi grew the same as on plates containing medium that was not mixed with any cultivation supernatant (control). All plant pathogenic fungi grew slower on plates containing sorbicillinol and the derived sorbicillinoids (i.e., supernatant of cultivation of *T. reesei* strains QM6a and Re*ypr1*). Notably, the growth inhibiting effect seems to be in direct relation to the amount of sorbicillinoids in the plate. We observed a less pronounced inhibition of growth of *A. alternata*, *B. cinerea*, and *R. solani* on plates with the supernatant of Δ*sor4*, which contains dihydrosorbicillinols and only small amounts sorbicillinol and no complex sorbicillinoids (**Figures 4**–**6**). *F. oxysporum* was not affected by the Δ*sor4* supernatant (**Figure 7**). Interestingly, the mycelium of *R. solani* had a more compact and organized appearance on Δ*sor4* supernatant than on the other plates. The growth of *A. alternata* is only marginally inhibited on Δ*sor4* supernatant, but sporulation is augmented compared to the control plate and Δ*ypr1* (**Figure 7**).

We were also interested, whether the sorbicillinoids might also have antibacterial activities. To this end, we used the supernatants of QM6a, Δ*ypr1*, and Re*ypr1* cultivated for 48 h on glucose in an agar diffusion assay against strains of *E. coli*, *A. baumanii*, *S. aureus*, and MRSA. As control, we used medium, which had not been inoculated with any *T. reesei* strain. None of the culture supernatants had a growth inhibiting effect on any of the tested bacteria (not shown).

### Sorbicillinoids Influence the Competition with Plant Pathogenic Fungi

**FIGURE 8:**
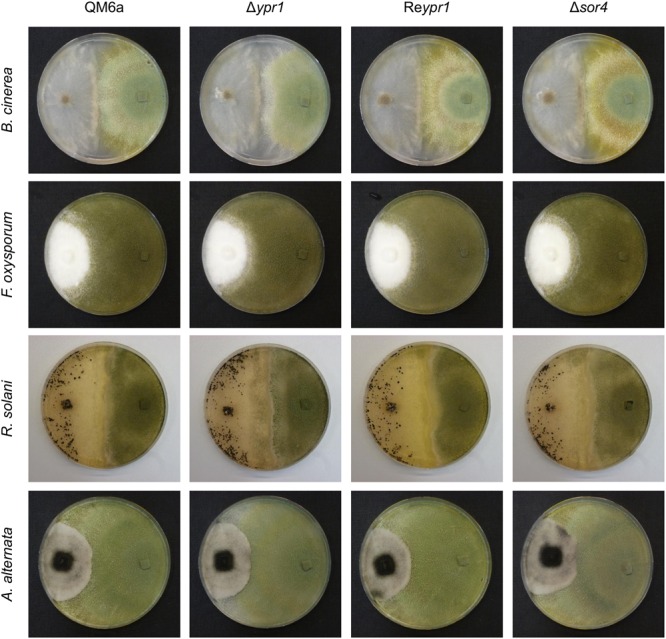
Influence of *T. reesei* sorbicillinoids on direct fungal confrontations. The *T. reesei* strains (indicated on top) and plant pathogenic fungi (indicated on the left) were inoculated on potato dextrose agar plates at opposite sides (*T. reesei* on the right, the plant pathogenic fungi on the left) using equal pieces of overgrown potato dextrose agar, and incubated at room temperature. Pictures were taken after 2 days after initial contact in the case of *B. cinerea*, and after 3 weeks in the case of *F. oxysporum, R. solani*, and *A. alternata*.

Next, we aimed to test whether the growth limiting properties of the sorbicillinoids might support *T. reesei* in confrontation with other fungi. To this end, we performed confrontation plate assays using the plant pathogenic fungi *A. alternata*, *B. cinerea*, *F. oxysporum*, and *R. solani* and the following *T. reesei* strains: the wild-type QM6a, the sorbicillinoid non-producer Δ*ypr1*, the sorbicillinoid hyper-producer Re*ypr1*, and Δ*sor4* which has a different sorbicillinoid spectrum [i.e., dihydrosorbicillinols, small amounts of sorbicillinol (**Figures 4**, **5**), but no other sorbicillinoids (**Figures 4**, **6**)]. Interestingly, *T. reesei*Δ*sor4* produced as much, or even more, yellow pigments as QM6a on the plates (**Figure 8**), in contrast to liquid cultures (**Figure 3A**). We comment on this in the Section “Discussion.” However, we observed no differences at all among the four *T. reesei* strains in confrontation to *A. alternata* (**Figure 8**). In confrontation with the other three fungi, we observed only subtle differences among the four *T. reesei* strains. Against *B. cinerea*, Δ*ypr1* grows weaker in vicinity to *B. cinerea* than the other strains at early time points. In confrontation with *F. oxysporum*, Δ*ypr1* gets stronger overgrown than QM6a, while Re*ypr1* is able to resist *F. oxysporum* better than the other strains. Notably, the mycelium of *F. oxysporum* overgrowing Δ*sor4* has a more structured and denser morphology than on the other *T. reesei* strains. Surprisingly, the sorbicillinoid non-producer Δ*ypr1* most strongly overgrows *R. solani* in comparison to the other *T. reesei* strains (**Figure 8**).

## Discussion

### On the Biosynthesis Pathway of Sorbicillinoids, 5-Hydroxyvertinolide, and the Roles of Sor4 and ACRE_048140

Previously, *P. chrysogenum* SorC was demonstrated to oxidize sorbicillin and 2′,3′-dihydrosorbicillin to sorbicillinol and 2′,3′-dihydrosorbicillinol, respectively, *in vitro* (Fahad et al., 2014). Based on these results, *in silico* analyses of the PKS SorA and SorB, and previous data from metabolite identifications and radio-labeled feed experiments, the authors proposed the model described in **Figure 1**. An earlier model had claimed that oxosorbicillinol was a key intermediate for sorbicillinol synthesis via a hypothetical compound, and that sorbicillin was derived from sorbicillinol (Abe et al., 2002). Our data support the newer model proposed by Fahad et al. (2014), because we detected the highest amounts of sorbicillin at the earliest time point, and because we observed the accumulation of oxosorbicillinol only after sorbicillinol had been built, timely and hierarchically (**Figures 4**, **6**).

The model of Abe et al. (2002) also suggested that 5-hydroxyvertinolide was derived from the same hypothetical compound as sorbicillinol. Another, previous model proposed that epoxysorbicillinol could be converted into 5-hydroxyvertinolide (Sperry et al., 1998). Our observations do not support any of these two models, because we detect 5-hydroxyvertinolide already at early time points in dependence of sorbicillinol (**Figure 4**). We consider 5-hydroxyvertinolide, as well as the unknown, early metabolite K_307 (**Table 2**), to be (chemically and/or enzymatically) derived from sorbicillinol because they are barely present in Δ*sor4* (**Figure 4**).

**FIGURE 9:**
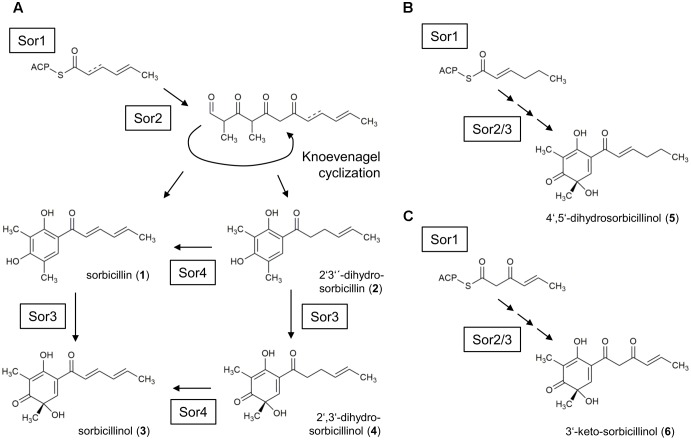
Model for the sorbicillinol biosynthesis pathway in *T. reesei*. **(A)** The basic model is identical to the earlier proposed model by Fahad et al., 2014 (compare **Figure 1**). Briefly, the PKS Sor1 iteratively combines three acetyl units and the growing chain is modified by the ketoacyl reductase subunit, and optional by the enoyl reductase subunit in the second cycle. The polyketide is handed over to the PKS Sor2, which adds three more acetyl units, and two methyl groups. Sor2 releases an aldehyde, which undergoes spontaneous cyclization resulting in the formation of sorbicillin (**1**) or 2′,3′-dihydrosorbicillin (**2**). Next, the FAD-dependent monooxygenase Sor3 transforms sorbicillin or 2′,3′-dihydrosorbicillin to sorbicillinol (**3**) or 2′,3′-dihydrosorbicillinol (**4**). Further, Sor4 might reduce 2′,3′-dihydrosorbicillin (**2**) to sorbicillin (**1**) and/or 2′,3′-dihydrosorbicillinol (**4**) to sorbicillinol (**3**). The obtained data from our study (**Figures 4**–**6**) suggest that the main product of Sor1/2 is in fact 2′,3′-dihydrosorbicillin. **(B)** Assumed that the enoyl reductase subunit of Sor1 was active during the first cycle, 4′,5′-dihydrosorbicillinol (**5**) would have been synthesized by Sor2 and Sor3. **(C)** Assumed that the ketoacyl reductase subunit of Sor1 was not active during the third cycle, Sor2 and Sor3 would have assembled 3′-keto-sorbicillinol (**6**).

However, we would like to extend the model proposed by Fahad et al. (2014) on the biosynthesis of sorbicillinol in *T. reesei*. We observed the accumulation of the two compounds, J_251 and F_251, that were both annotated to dihydrosorbicillinol (**Table 2**), but only small amounts of sorbicillinol in *T. reesei* Δ*sor4* (**Figures 4**, **5**). Notably, we did not detect the dihydrosorbicillinols in any other strain. We speculate, that the early arising compound J_251 might be 2′,3′-dihydrosorbicillinol. This assumption is based on the already existing model, which proposes that 2′,3′-dihydrosorbicillinol is synthesized in parallel to sorbicillinol (Fahad et al., 2014) (**Figure 1**). Sor4 is an FAD-binding dehydrogenase according to a conserved domain analysis. These enzymes are able to reduce alkanes to alkenes, because FAD has such a high reduction potential that it can accept two electrons and two protons simultaneously. The accumulation of dihydrosorbicillinol in the absence of Sor4 suggests that Sor4 might reduce the double-bond in the linear side-chain of 2′,3′-dihydrosorbicillinol (**4**). This implies that the main product of the PKS-cascade and the cyclization reaction is in fact 2′,3′-dihydrosorbicillin (**2**), which is oxidized to 2′,3′-dihydrosorbicillinol (**4**) by Sor3. Finally, Sor4 might reduce 2′,3′-dihydrosorbicillinol (**4**) to sorbicillinol (**3**). Alternatively, Sor4 might as well reduce 2′,3′-dihydrosorbicillin (**2**) to sorbicillin (**1**) before it is oxidized to sorbicillinol (**3**) by Sor3 in a final step (**Figure 9A**). Of course, the two alternative pathways might occur simultaneously.

F_251, the second dihydrosorbicillinol in the Δ*sor4* supernatant (**Figure 5**), could be 2′,5′-dihydrosorbicillinol or 4′,5′-dihydrosorbicillinol or another unknown isomer; 2′,5′-dihydrosorbicillin was previously identified as a product of chemical hydrogenation of sorbicillin (Trifonov et al., 1983). If F_251 was 2′,5′-dihydrosorbicillinol, its occurrence could be explained by a chemical conversion. This speculation is supported by the fact that F_251 accumulates over time (**Figure 5**). Alternatively, it could be another unknown isomer that occurs by chemical isomerization. If F_251 was 4′,5′-dihydrosorbicillinol, its occurrence could be explained by the implied sloppy mode of action of SorA/1, as already discussed by Fahad et al. (2014). The PKS SorA/1 contains, next to the core acyl transferase subunit, a presumably non-functional methyl transferase subunit, a ketoacyl reductase subunit, and an enoyl reductase subunit. The ketoacyl reductase subunit reduces the beta-carbonyl of the growing polyketide to a hydroxyl group. The enoyl reductase subunit reduces a double to single bond. Depending on, whether, and during which cycle of chain elongation the enoyl reductase subunit is active, sorbicillinol, 2′,3′- or 4′,5′-dihydrosorbicillinol would be synthesized in the end (compare **Figures 9A,B**, compounds **3**, **4**, and **5**).

Further, if we speculate that the ketoacyl reductase subunit of Sor1 was not active during a cycle of polyketide elongation, a sorbicillinol derivate containing a keto-group would be synthesized ultimately (**Figure 9C**, compound **6**). This hypothetical compound has the same empiric formula as oxosorbicillinol and epoxysorbicillinol, and might be compound F_265 (**Table 2**) that was detected in all sorbicillinoid producing strains (**Figure 5**). Its higher levels in Δ*sor4* could be explained by a feedback of the accumulating dihydrosorbicillinol on Sor1.

The amounts of early metabolites were nearly equal in the wild-type-like QM6a and the strain A4814, which bears the hydrolase ACRE_048140 from *A. chrysogenum* (**Figure 4**). We detected smaller amounts of the late metabolites in A4814 than in QM6a (**Figure 6**), but we consider this to be the result of lower sorbicillinol levels in strain A4814 (**Figure 4**) because these metabolites are derived from sorbicillinol. Therefore, we conclude that the hydrolase ACRE_048140 did not influence the sorbicillinoid biosynthesis in *T. reesei*.

### On the Growth-Limiting Properties of Sorbicillinoids

During the growth experiment of the recombinant strains, we observed that the growth rate seems to be negatively related to the biosynthesis of sorbicillinoids (**Figure 3**). The strains, which do not produce sorbicillinoids, Δ*ypr1* and Δ*sor1*, grew better than the wild-type-like strains QM6a and A4814, whereas the sorbicillinoid hyper-producer Re*ypr1* accumulated less biomass. Interestingly, Δ*sor3* and Δ*sor4* grew equally well as the wild-type-like strains, although they do not produce mature sorbicillinoids. In Δ*sor3* the biosynthesis pathway is disrupted after the cyclization of the released aldehydes (**Figure 1**), implying that intracellular sorbicillin and dihydrosorbicillin are built. In Δ*sor4* dihydrosorbicillinol accumulates, very small amounts of sorbicillinol are synthesized, and no late sorbicillinoids are produced (**Figure 6**). Therefore, the reason for the growth limitation is likely to be either the metabolic burden or the intracellular presence of sorbicillin and dihydrosorbicillin (**Figure 9**). It cannot be excluded that the extracellular sorbicillinoids also have a growth limiting effect on *T. reesei*. Probably, all three factors contribute to some extents to the observed growth restrictions.

During the confrontation assays on the plates, we observed that Δ*sor4* turned the medium more intensely yellow than the wild-type-like QM6a (**Figure 8**), but it was the opposite in liquid cultures (**Figure 3A**). Generally, we observed that the medium in plates became more yellow than in liquid cultures. We speculate that oxygen and/or light might degrade the sorbicillinoids. The strain Δ*sor4* produces predominantly dihydrosorbicillinols (**Figure 5**), which might be more susceptible to degradation by light and/or oxygen than sorbicillinol and the late sorbicillinoids. The presence of the dihydrosorbicillinols and the lack of the mature sorbicillinoids explain why Δ*sor4* did influence neither the morphology of the other fungi nor their growth rates.

The extracellular sorbicillinol and/or mature sorbicillinoids had a clear influence on the growth of other fungi (**Figure 6**). In direct fungal confrontations, the sorbicillinoids supported growth of *T. reesei* in presence of *B. cinerea* and *F. oxysporum*, however, subtle the effects were. In confrontation against *B. cinerea*, the radical-scavenging properties and/or the chemical reactivity of the sorbicillinoids might protect *T. reesei* from secreted compounds and/or proteins of *B. cinerea*. In confrontation against *F. oxysporum* and *R. solani*, the growth-inhibiting effects pose an obstacle for the stronger fungus. This is, the sorbicillinoids delayed *F. oxysporum* in overgrowing *T. reesei*, but they also hindered *T. reesei* from overgrowing *R. solani*. Taken together, the sorbicillinoids pose a possibility for *T. reesei* to maintain its already claimed territory in confrontation with other fungi, but it has to put up with their growth limiting effects.

## Author Contributions

CD constructed the plasmids, the deletion strains, performed the growth experiment, and the fungal confrontation assays, performed data analysis, co-drafted the manuscript, and was involved in the study design. FG-C performed the LC-MS analyses and performed data analysis. TM-d-S constructed the strain A4814. H-JB performed the agar diffusion assay. AD performed data analysis. RM was involved in the study design. AM-A co-drafted the manuscript and was involved in the study design.

## Conflict of Interest Statement

The authors declare that the research was conducted in the absence of any commercial or financial relationships that could be construed as a potential conflict of interest.
