# Supplementary material for: In Vivo Study of the Sorbicillinoid Gene Cluster in Trichoderma reesei
Source: Front Microbiol. 2017 Oct 20;8:2037. doi: 10.3389/fmicb.2017.02037 (PMC5654950; doi:10.3389/fmicb.2017.02037)
Supplement: Supplementary file 1 [file Data_Sheet_1.pdf]

Supplementary Material

In vivo study of the sorbicillinoid gene cluster in *Trichoderma reesei*

Christian Derntl, Fernando Guzmán-Chávez, Thiago M. Mello-de-Sousa, Hans-Jürgen Busse, Arnold J.M. Driessen, Robert L. Mach, Astrid R. Mach-Aigner*

Correspondence: Dr. Astrid Mach-Aigner: astrid.mach-aigner@tuwien.ac.at

Table S1. Primers used throughout this study

Primer name	Sequence (5' – 3')	Usage
sor1_5fwd-NotI	GCGGCCGCGTGCTGATAAAGGACAAGATG	Cloning
sor1_5rev-BglII	AGATCTCTGAATCTAGAGGCTGGGAAC	
sor1_3fwd-ClaI	ATCGATAGATCCGCAATTCATCCTCG	
sor1_3rev-HindIII	AAGCTTGATGAATACTGACGGACGAGG	
sor3_5fwd-NotI	GCGGCCGCGTCAAGTACCTAGGTATGCTTC	
sor3_5rev-BspEI	TCCGGACGTTGGGAGTAAAGTGTATGCC	
sor3_3fwd-XbaI	TCTAGACGAGATCCTAGAACGAAGCC	
sor3_3rev-BglII	AGATCTCACTGGGCTGTTAGCATTGTC	
sor4_5fwd-HindIII	AAGCTTGTAAGATCGGACCAGTGGCTG	
sor4_5rev-BcuI	ACTAGTGACTGCGATTCCGAAGGC	
sor4_3fwd-BspEI	TCCGGAGTGGCGAATTGCTAACAAGAC	
sor4_3rev-NotI	GCGGCCGCCAATAGGTATCCTGCCTTG	
P_{sor3}_fwd-BspEI	TCCGGACAGGCGTGGAAGGATAGC	
P_{sor3}_rev-SpeI	ACTAGTCTTGTTACTGGCTGTAGGAC	
ACRE_048140_fwd-SpeI	ACTAGTATGACCATCCCGGGCG	
ACRE_048140_rev-BamHI	GGATCCTTAACCTCGAGTCGCATTGATCC	
pyr4_Prev	GATAATACCTACCCAGTCAGACC	Genomic characterization
pyr4_Tfwd	GCAGCGTAACATCTTCAGAGC	
sor1_test5	GCTTCACACAGGGATGGCC	
sor1_test3	GTGACGATGACGGTTTTGCC	
sor3_test5	CGATGAGTCTGTATTGAAGGG	
sor3_test3	CCAAGAGGACGGCTGAATC	
sor4_test5	GGACTATCAAAAGGGACCGG	
sor4_test3	CAAAAGCCATGCACCGAC	
5pyr4_fwd2	CCAGACGGTGATTCACATATACG	
Tpyr4_rev2	ACAAGGACTGAGGATGTTCGG	
pyr4_3f	AGACGAGGACCAGCAGACC	

Table S1. Primers used throughout this study (continued)

Primer name	Sequence (5' – 3')	Usage
ypr1_q1f	GTTCTACACACGACTTCCCATG	
ypr1_q1r	CCAGCCACTGATGTCGTATCC	
sor1_q1f	GGCCTTTGTTCTTCATGACTCC	
sor1_q1r	GTGAGCCAAGGCATCTTCG	
sor3_q1f	CTTCGTCTTGAGTGTTCCTCTG	RT-PCR
sor3_q1r	GGCAGCAACGATATAAGCGAG	
sor4_q1f	CCTGGTAGTGAGAAACACGG	
sor4_q1r	GGCCAACAGTCGGACATATC	
ACRE_048140_q1f	CACAAACTCGCGCATCTTCAG	
ACRE_048140_q1r	ATGACCACGGCGTTGATCCAG	

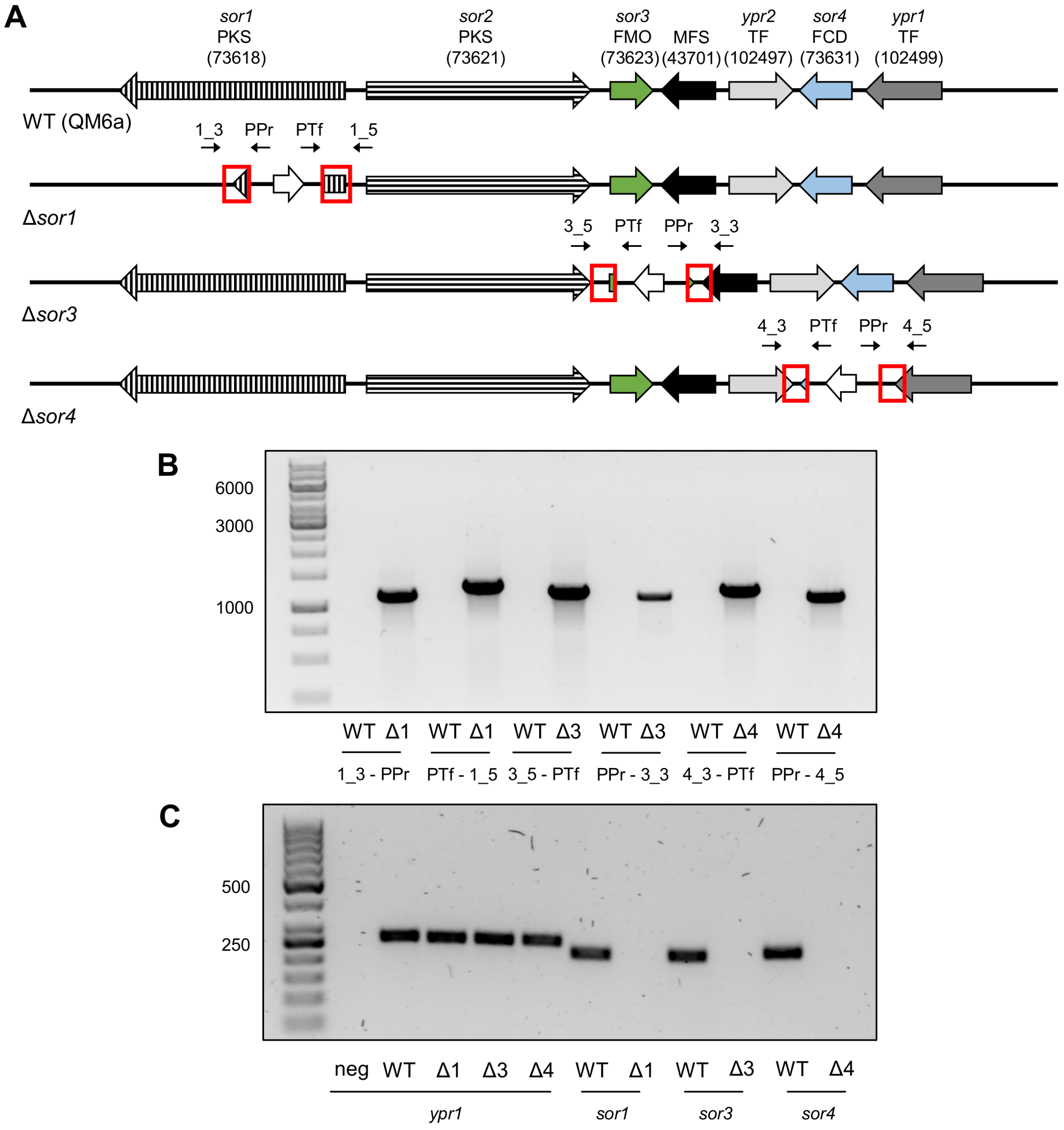

Figure S1. Deletion of *sor1*, *sor3*, and *sor4* in *T. reesei*

(**A**) The uridine auxotrophic strain Δ*pyr4*, which contains the wild-type gene cluster, like the parent strain QM6a, was transformed with the plasmids pCDΔsor1, pCDΔsor3, or pCDΔsor4. They bear the deletion cassettes to replace the genes *sor1*, *sor3*, or *sor4* with the marker *pyr4* (white arrow) via homologous recombination resulting in the strains Δ*sor1*, Δ*sor3*, and Δ*sor4*. Arrows indicate the genes of the cluster. Flanking regions used for homologous recombination are indicated by red boxes. Orientation, distance, and size are to scale. PKS, polyketide synthase; FMO, FAD-dependent monooxygenase; MFS, transporter of the multifacilitator superfamily; TF, transcription factor; FCD, FAD/FMN-containing dehydrogenase. Protein IDs are given in brackets. Small arrows represent

Figure S1. Deletion of *sor1*, *sor3*, and *sor4* in *T. reesei* (continued)

approximate position of the indicated primers used for genomic characterization. (**B**) PCRs were performed to test the successful integration of the deletion cassettes using chromosomal DNA from Δ*pyr4* (WT), Δ*sor1* (Δ1), Δ*sor3* (Δ3), and Δ*sor4* (Δ4) as template and the indicated primers. 1_3, sor1_test3; PPr, pyr4_Prev; PTf, pyr4_Tfwd; 1_5, sor1_test5; 3_5, sor3_test5; 3_3, sor3_test3; 4_3, sor4_test3; 4_5, sor4_test5. (**C**) A RT-PCR analysis was performed to test for the presence or absence of the indicated transcripts in the strains Δ*pyr4* (WT), Δ*sor1* (Δ1), Δ*sor3* (Δ3), and Δ*sor4* (Δ4). Water was used as template in the negative control (neg).

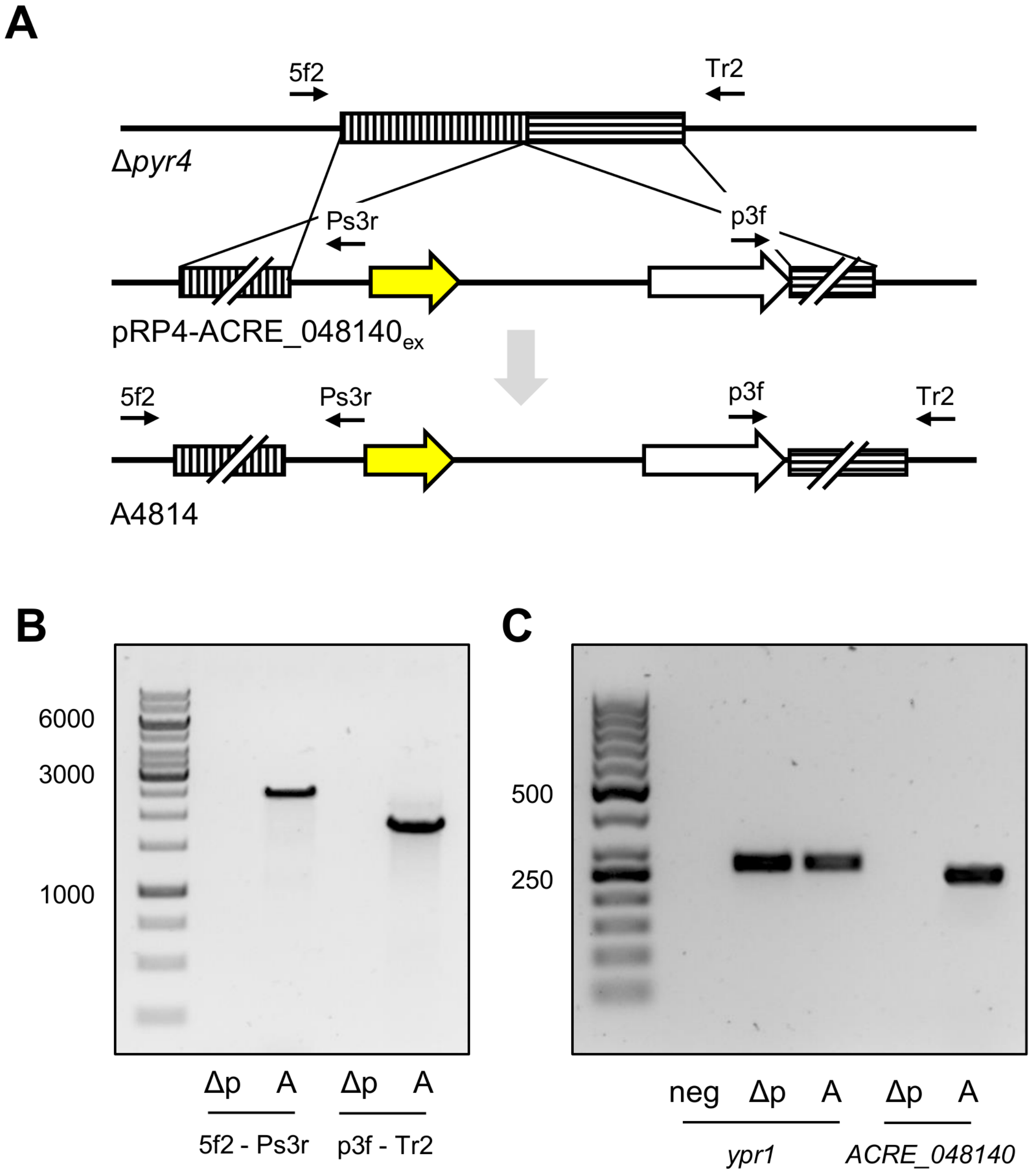

Figure S2. Insertion of ACRE_048140 expression cassette

(**A**) The uridine auxotrophic strain Δ*pyr4* was transformed with the plasmid pRP4-ACRE_048140$_{ex}$, which contains the expression cassette for the hydrolase ACRE_048140 (yellow arrow) and the *pyr4* gene (white arrow) between the flanking regions (hatched boxes) of *pyr4*. Upon homologous recombination, the gene encoding for ACRE_048140 is integrated at the *pyr4* locus and the prototrophy is re-established, resulting in the strain A4814. Orientation, distance, and size are to scale. The grey arrows indicate the homologous recombination event. Small arrows represent binding approximate position of the indicated primers used for genomic characterization in (**B**). PCRs were performed to test for the successful integration of the expression cassettes using chromosomal DNA from Δ*pyr4* (Δp), and A4814 (A) as template and the indicated primers. 5f2, 5pyr4_fwd2; Ps3r, P$_{sor3}$_fwd-BspEI; p3f, pyr4_3f; Tr2, Tpyr4_rev2. (**C**) A RT-PCR was performed to test for the presence or absence of the indicated transcripts in the strains Δ*pyr4* Δ*pyr4* (Δp), and A4814 (A). Water was used as template in the negative control (neg).
